# Miscellaneous

**Published:** 1891-04

**Authors:** 


### MISCELLANEOUS.

### TOUGHENING LAMP CHIMNEYS.

A correspondent of a trade journal lately asked, "Has the tendency of hardened glass to break spontaneously yet been overcome?" The reply to the inquiry states that "this great difficulty with hardened glass has been overcome by Frederick Siemens, by placing the hardened cold plates in boiling water, and allowing them to cool very slowly; and that he has made some very interesting experiments before the Berlin Polytechnic Society, showing the power of resistance of such plates. A plate about 8 in. square and more than an inch thick was supported at its corners and on it was dropped a weight of more than 8 lb., from different heights, up to 6 ft., without its breaking. A second and a third test were made, the third plate breaking under a weight of 1 lb. 6 oz., falling 11½ ft., while the same weight falling 2 ft. broke an ordinary plate of the same dimensions. In applying this principle of toughening the glass to lamp chimneys we should prefer to place them at first in cold, rather than boiling, water. If the water is heated slowly to the boiling point, kept there for some time, and then cooled as above, cheap chimneys can be used for months, or until some accident befalls them. We can testify from experience that the plan is a successful one.

THE first newspaper ever issued in the world was published in Venice, in 1531, eighty-one years after Faust invented printing. It was called *The Gazetta.* The first newspaper printed in England was *The English Mercurie*, published July 28, 1588, in London, by Christopher Barker.

### TAKE TIME TO EAT.

The opinion that hurry in eating is a prolific cause of dyspepsia is founded on common observation. The ill results of "bolting" the food have been attributed to the lack of thorough mastication, and to the incomplete action of the saliva upon the food. Two-thirds of the food which we eat is starch, and starch cannot be utilized by the system as food until it has been converted into sugar, and this change is principally effected by the saliva. But there is a third reason why rapidity of eating interferes with digestion. The presence of the salivary secretion in the stomach acts as a stimulus to the secretion of the gastric juice. Irrespective of the mechanical function of the teeth, food which goes into the stomach incompletely mingled with saliva, passes slowly and imperfectly through the process of stomach digestion. Therefore, as a sanitary maxim of no mean value, teach the children to eat slowly—and in giving this instruction by example, the teacher, as well as the pupil, may receive a benefit.—*Sanitary Inspector.*

### A STARTLING CLASS EXPERIMENT.

Dr. Koucharsky was lecturing to his class of medical students in St. Petersburg a few weeks ago, when, at the close of his remarks on the subject of acids, he poured into a glass, from a small bottle, a quantity of liquid, and said : "Attention, gentlemen ! In two minutes you will see a man die. Good-bye to you all !"—and drank the liquid. He took his watch from his pocket, for some seconds looked at it, and then fell to the floor lifeless, to the horror of his audience, who had not the slightest idea of his intent. Antidotes were then applied, but in vain.

### THE AGE AT WHICH TO WED.

M. Korosi, of the Hungarian Academy of Sciences has collected about thirty thousand data, and has come to the following conclusion: Mothers under twenty years of age, and fathers under twenty-four, have children more weakly than parents of riper age. Their children are more subject to pulmonary diseases. The healthiest children are those whose fathers are from twenty-five to forty years of age, and whose mothers are from twenty to thirty years old. M. Korosi says, and most medical men indorse this view, that the best marriages are those in which the husband is senior to the wife.

THE DOLLAR SIGN ($) is not a monogram of "U. S.," but dates from the days when the transfer was made from Spanish to American dollars, and accounts were kept equally in dollars and reals. Thus : one dollar ‖ eight reals (American and Spanish parallel accounts). Later the 8 was placed between the cancellation mark |8| ; then the perpendicular lines crossed the 8, and finally the 8 shaded into an S, and, combined with the cancellation line, evolved the present sign ($).

FOR BRAIN FAG use Horsford's Acid Phosphate. Dr. W. H. Fisher, Le Sueur, Minn., says: "I find it very serviceable in nervous debility, sexual weakness, brain fag, excessive use of tobacco, as a drink in fevers, and in some urinary troubles. It is a grand good remedy in all cases where I have used it."

### SMALL THINGS OF GREAT ACCOUNT.

The thread of a silkworm is so small that an average of forty-two of them are twisted together to form a thread of common sewing silk; that of the spider are many diameters smaller. Two drachms of spider-web by weight would, if stretched into a straight line, reach from London, England, to Edinburgh, Scotland, a distance of over 400 miles.

In sour paste, the milt of a codfish, or even in water in which vegetables have been infused, the microscope discovers animalculi so small that millions of them would not equal the size of a grain of wheat. And yet nature, with a singular prodigality, has supplied many of these with organs as complete as those of a whale or an elephant. In a single ounce of such matter there are more living creatures than there are human beings on the face of the globe.

A grain of carmine or half a grain of analine will tinge a hogshead of water so that a strong microscope will detect coloring matter in every drop.

A grain of musk will scent a room for twenty years, and at the end of that time will not show that it has diminished in the least.

The organs of smell in the turkey vulture and carrion crow are so delicate that they can scent their food for a distance of forty miles.

Gold-beaters, by hammering, can reduce gold leaves to such minute thinness that 282,000 must be laid upon each other to produce the thickness of an inch. Yet each leaf is so perfect and free from holes that one of them laid on any surface, as in gilding, gives the appearance of solid gold. They are so thin that if formed into a book 1,500 would only occupy the space of a single leaf of book paper. A single volume of a gold leaf book one inch in thickness would have as many pages as an entire library of 1,500 volumes of common books, even though the volumes average 400 pages each!

It has been drawn into wire so fine that twenty-seven of them twisted together could be inserted into a hollow of a hair.

The ruled lines of the glass measure of the microscopist are drawn so fine that more than five hundred of them occupy a space no greater than the edge of an ordinary book-leaf. Don't ask us how this is done, but find out some other way.

---

### A CURE FOR DIPHTHERIA.

The following remedy was discovered in Germany and is said to be the best known. At the first indication of diphtheria in the throat of a child make the room close; then take a tin cup and pour into it a quantity of tar and turpentine, equal parts. Then hold the cup over a fire so as to fill the room with fumes. The little patient on inhaling the fumes, will cough up and spit out all the membranous matter, and the diphtheria will pass off. The fumes of the tar and turpentine loosen the matter in the throat, thus affording the relief that has baffled the skill of physicians.

---

Read the advertisement of Herba Vita, the remarkable preventive of disease. No alcohol used in its preparation.

### BOARD OF HEALTH.

The annual report of the Board of Health recently submitted to Mayor Grant, shows the number of deaths recorded during the past year to be 40,103, an excess over 1889 of 424. In the last decade the death rate has decreased from 26.41 to 24.58, and President Wilson claims that New York is the healthiest city in the world.

The number of tenement houses in the city is 37,316, and 1,259,788 people live in them, as the report says. There were 39,250 births last year, an increase of 9,000 over 1889. There were 14,992 marriages recorded.

The milk inspectors made 244 arrests and secured 14 convictions, from which a very large amount was collected in fines.

